# Involvement of CCR6/CCL20/IL-17 Axis in NSCLC Disease Progression

**DOI:** 10.1371/journal.pone.0024856

**Published:** 2011-09-15

**Authors:** Sophie Kirshberg, Uzi Izhar, Gail Amir, Jonathan Demma, Fiona Vernea, Katia Beider, Zippora Shlomai, Hanna Wald, Gideon Zamir, Oz M. Shapira, Amnon Peled, Ori Wald

**Affiliations:** 1 Goldyne Savad Institute of Gene Therapy, Hadassah University Hospital, Jerusalem, Israel; 2 Department of Cardiothoracic Surgery, Hadassah University Hospital, Jerusalem, Israel; 3 Department of Pathology, Hadassah University Hospital, Jerusalem, Israel; 4 Laboratory for Surgical Research, Hadassah University Hospital, Jerusalem, Israel; 5 Department of Surgery, Hadassah University Hospital, Jerusalem, Israel; University of Texas Southwestern Medical Center at Dallas, United States of America

## Abstract

**Objectives:**

Autocrine and paracrine chemokine/chemokine receptor-based interactions promote non-small-cell-lung-cancer (NSCLC) carcinogenesis. CCL20/CCR6 interactions are involved in prostatic and colonic malignancy pathogenesis. The expression and function of CCL20/CCR6 and its related Th-17 type immune response in NSCLC is not yet defined. We sought to characterize the role of the CCL20/CCR6/IL-17 axis in NSCLC tumor growth.

**Methods:**

A specialized histopathologist blindly assessed CCL20/CCR6 expression levels in 49 tissue samples of NSCLC patients operated in our department. Results were correlated to disease progression. Colony assays, ERK signaling and chemokine production were measured to assess cancer cell responsiveness to CCL20 and IL-17 stimulation.

**Results:**

CCL20 was highly expressed in the majority (38/49, 77.5%) of tumor samples. Only a minority of samples (8/49, 16.5%) showed high CCR6 expression. High CCR6 expression was associated with a shorter disease-free survival (P = 0.008) and conferred a disease stage-independent 4.87-fold increased risk for disease recurrence (P = 0.0076, CI 95% 1.52–15.563). Cancerous cell colony-forming capacity was increased by CCL20 stimulation; this effect was dependent in part on ERK phosphorylation and signaling. IL-17 expression was detected in NSCLC; IL-17 potentiated the production of CCL20 by cancerous cells.

**Conclusion:**

Our findings suggest that the CCL20/CCR6 axis promotes NSCLC disease progression. CCR6 is identified as a potential new prognostic marker and the CCL20/CCR6/IL-17 axis as a potential new therapeutic target. Larger scale studies are required to consolidate these observations.

## Introduction

Primary carcinoma of the lung is the second most frequent (12%) cancer worldwide, and is the leading cause of cancer related death. NSCLC (mainly lung adenocarcinoma) accounts for nearly 80% of cases. Lung cancer is linked to a long history of smoking and to its accompanying chronic inflammatory response [1], [2], [3]. Chemokines - a family of chemotactic cytokines, are master regulators of immune cell trafficking in the body [4]. Chemokines interact with seven trans-membrane-G-protein-coupled receptors to exert their effects [4]. Distinct immune cell subtypes express specific repertoires of chemokine receptors, which guide their trafficking, retention and function in target organs [5]. A variety of tumor cells express chemokine and chemokine receptors [6]. Activation of the chemokine\chemokine receptor axis within tumors induces autocrine and paracrine loops promoting tumor growth and angiogenesis and subverting antitumor immune response [6], [7].

Distinct cytokine and chemokine/chemokine receptors characterize specific types of immune responses [8]. IFN-g and CXCR3 are characteristic of Th-1-type immune response while IL-4, 5, 13 and CCR4, CCR10 are characteristic of Th-2-type immune response [9]. Th-17-type immune response is linked to CCL20 and CCR6. Th-17 cells contribute to the eradication of extracellular bacterial infections and also play a major role in autoimmunity [10], [11]. The involvement of Th-17 response in malignant diseases remains unclear [12]. Ovarian cancer cells were shown to promote the differentiation of Th-17 cells [13]. Accumulation of Th-17 cells in hepatocellular carcinoma was linked to a worse prognosis [14].

The chemokine/chemokine receptor pair CCL20/CCR6 is a key player in lung immunity [15]. CCL20/CCR6 is involved in the pathogenesis of smoke-related chronic inflammatory conditions such as chronic obstructive pulmonary disease and interstitial lung fibrosis [16], [17]. Activation of the CCL20/CCR6/IL-17 axis promotes the eradication and recovery of the lung following Klebsiella pneumoniae infection [18]. CCL20/CCR6 interactions have recently been linked to the propagation of several malignancies such as prostate, hepatic and pancreatic carcinomas, raising the possibility that this axis also participates in lung carcoinogenesis [19].

The expression, regulation and function of CCL20/CCR6/IL-17 in NSCLC have not been characterized thus far. We sought to characterize the role of the CCL20/CCR6/IL-17 axis in NSCLC tumor growth.

## Materials and Methods

### Tissue collection and patient-specific clinical data

Fresh human lung and tumor specimens were obtained from patients (n = 20) undergoing complete resection of early stage NSCLC (clinical stage IA-IIB) who had not received preoperative chemotherapy or radiotherapy to exclude confounding effects. Histological sections were prepared from these samples and an experienced pathologist (GA) confirmed the histopathological diagnosis. These tissues were used for the various experiments described in this manuscript. In order to assess the correlation between CCL20/CCR6 expression and lung adenocarcinoma disease progression, we additionally collected 49 paraffin-embedded tissue sections of lung adenocarcinoma tumors (clinical stage IA-IIB) that were removed from patients in our department. The study period was January, 2000 to September, 2010. Patients did not receive preoperative chemotherapy or radiotherapy to exclude confounding effects. All patients underwent an extensive sampling of mediastinal lymph nodes. An experienced pathologist (GA) reassessed the slides to re-confirm the diagnosis. Clinical data (survival, time to disease recurrence and pathological staging) of these patients was reviewed. The Hadassah Hospital Ethics Committee approved the human component of the study. A written informed consent was obtained from all participants involved in this research.

Assessment of CCR6 expression in lung adenocarcinoma and correlation analysis to pathologic stage of disease were also done using the Biomax tissue array: BC041115, which is a lung carcinoma and normal tissue array (Biomax US. 1100 Taft St., Rockville, MD 20850, USA).

### Immunohistochemistry of CCL20 and CCR6

Antigen retrieval was performed in EDTA buffer for 20 minutes in microwave. Sections were stained with the anti-human CCR6 monoclonal antibody (R&D Systems, Minneapolis, MN 55413, USA) - concentration 10 mg/ml, or with anti-human CCL20 polyclonal antibody (PeproTech EC, London, UK), - concentration of 20 mg/ml. Next, the sections stained for CCL20 were incubated with diluted 1∶1000 biotinylated goat-antirabbit antibody (Jackson ImmunoResearch), for 30 minutes and thereafter with horseradish peroxidase - conjugated streptavidin (Zymed Laboratories, San Francisco, CA,USA) for 30 minutes. The sections stained for CCR6 or CCL20 were incubated with secondary anti-mouse horseradish peroxidase-conjugated antibody (DakoCytomation, Glostrup, Denmark) for 30 minutes at room temperature. 3-amino-9-ethylcarbazole (AEC) was used for color development, and sections were counterstained with hematoxylin. Negative control sections were stained with either no primary antibody (PBS) or with isotype-matched control antibody.

### Histological evaluation of CCL20/CCR6 staining

Two experienced pathologists blindly assessed tissue sections for the percentage of tumor cells staining positive for CCL20 and CCR6. Samples were divided into four categories: 1. 0–25% of tumor cells stained positive, 2. 25–50% of tumor cells stained positive, 3. 50–75% of tumor cells stained positive, 4. 75–100% of tumor cells stained positive. High expression of the chemokine or receptor was defined if more than 50% of tumor cells stained positive.

Analysis of the Biomax lung cancer tissue array was done as follows: CCR6 staining intensity was scored from 0 to 3 (0 – no staining, 1 – week intensity, 2 – medium intensity and 3 – high intensity); the percentage of CCR6 positive cells (four categories as described above) was also scored. For each sample, we calculated a CCR6 staining index: CCR6 index  =  (CCR6 Staining intensity * Percentage of CCR6 positive tumor cells)/100.

### ELISA assays of CCL20 and IL-17

Tumor and lung tissue homogenates were prepared on ice from weighed tissue samples. CCL20 levels in these homogenates were measured. Levels of CCL20 in the supernatant of NSCLC-derived cell lines (L3, L4 and L549) were measured in the presence or absence of IL-17 (25, 100, 1,000 ng/ml). IL-17 levels were measured in the supernatant of tumor-derived immune cells and in the supernatant of lung-derived immune cells in the presence or absence of anti OKT3 antibody. ELISA assays were performed with Quantikine kits according to the manufacturer's instructions (R&D Systems, Inc., Minneapolis, MN 55413, USA).

### Isolation of normal lung- and tumor tissue-derived lymphocytes

Isolation of normal lung- and tumor tissue-derived lymphocytes was done as previously described [20]. For activation experiments, 2*10^5^ cells were plated in each well in the presence or absence of (10 microgram/ml) anti CD3 antibody clone OKT3 (eBioscience); supernatant was collected after 5 days.

### NSCLC cell lines

The A549 – cell line was purchased from the ATCC (Rockville, MD, USA). Two primary NSCLC cell lines were generated in our laboratory as previously described; the L3 line was generated from lung adenocarcinoma and the L4 line from large cell carcinoma [21]. All cell lines were tested for mycoplasma contamination and were found to be negative.

### Fingerprinting and Tissue genotyping

In order to ascertain that our NSCLC-derived cell lines, L3 and L4, differ from each other and from the A549 ATCC cell line, we performed fingerprinting of these cells as described by Silva et al. [22]. We also performed tissue genotyping with the Applied Biosystems AmpFlSTR Identifiler plus kit, according to the manufacturer's protocol. The fingerprinting data are provided as supplementary material (1).

### Flow cytometry analysis of neoplastic cells

We performed flow cytometry analysis of 1.5X10^5^/ml of primary tumor-derived neoplastic cells (L3 and L4) and the NSCLC cell line A549. Cells were blocked with 1% human plasma for 15 minutes and then mixed with PE - conjugated anti-CCL20 monoclonal antibody clone 67310 or with monoclonal anti-CCR6 antibody, clone 53103.11 (R&D Systems, Inc., Minneapolis, MN 55413, USA) or with isotype control for 20. For CCL20 staining cells, the fixation and permeabilization method was performed prior to incubation with the antibody. Immunostained cells were analyzed by flow cytometry using the FACS Caliber Flow Cytometer (Becton Dickinson, Mountain View, CA, USA). Data were analyzed using the CellQuest software. (Version 3.3, Becton Dickinson).

We used the anti-CCR6 antibody clone 53103.11 in both flow cytometry and immunohistochemistry staining. To demonstrate the specificity of this clone for CCR6, we incubated fresh PBMC in the presence or absence of 20 ug/ml CCL20 for one hour and stained the cells for CCR6 or with isotype control antibody. Reduced CCR6 staining was observed in CCL20 treated cells; results are provided as supplementary material (Figure S2).

### Western blot of ERK and p-ERK

Western blot analysis of ERK and phosphorylated ERK was carried out as previously described and according to the manufacturer's recommendation [23]. In short, cells were stimulated with 500 ng/ml of CCL20 (R&D Systems Minneapolis, MN 55413, USA), and harvested at 0, 5, 15, 30 and 60 minutes. ERK and pERK detection was done (rabbit polyclonal antibodies specific for p-ERK, ERK - Santa Cruz, CA, USA) following the manufacturer's recommendations. Goat anti-rabbit horseradish peroxidase–conjugated antibodies were then used. Bands were scanned by a densitometer ImageMaster VDS-CL machine, (Pharmacia Biotech, Piscataway, NJ, USA).

### Colony assays

Agar base layer was prepared as follows: 45 ml of RPMI +12% FCS was mixed with 15 ml of RPMI X2 + 12% FCS and with 15 ml of 2.5% agar in double distilled water. Tumor cells were suspended in RPMI + 10% FCS. Cell suspension was mixed in a ratio of 1∶3 with the agar base solution. This mixture was then plated on top of a preformed solid agar base. CCL20, at the concentrations of 10 ng/ml, 50 ng/ml and 250 ng/ml, and the ERK inhibitor PD98059 at the concentration of 20 microliter/ml, were added to the mixture. Fourteen days later, the number of colonies was counted in ten different fields.

### PCR

The following primers were used:

CCL20 – sense ATGTGCTGTACCAAGAGTTT, antisense CAAGTCTGTTTTGGATTTGC.

CCR6 – sense CCATTCTGGGCAGTGAGTCA, antisense – AGCAGCATCCCGCAGTTAA

IL-17A – sense CAACCGATCCACCTCACCTT, antisense – GGCACTTTGCCTCCCAGAT

IL-17A receptor – sense GCGCCCAGACCAGAAGAG, antisense – CCCTTTAAGGTTGCGTAGAGTGA

b-actin – sense CCCTGGACTTCGAGCAAGAG, antisense – TCTCCTTCTGCATCCTGTCG

### Statistical Analysis

Data are expressed as mean ± standard error (SE) and as absolute values. Continuous data with normal distribution were analyzed using t-test. Time to disease recurrence was estimated non-parametrically by the Kaplan-Meier method. Cox regression analysis was performed to define the relevance of high CCR6 expression as a disease stage independent marker for disease recurrence. A p value of 0.05 was considered significant. Statistical analyses were performed using the SPSS software (IBM).

## Results

### Expression of CCR6/CCL20 in NSCLC tissue samples

**Figure 1:**
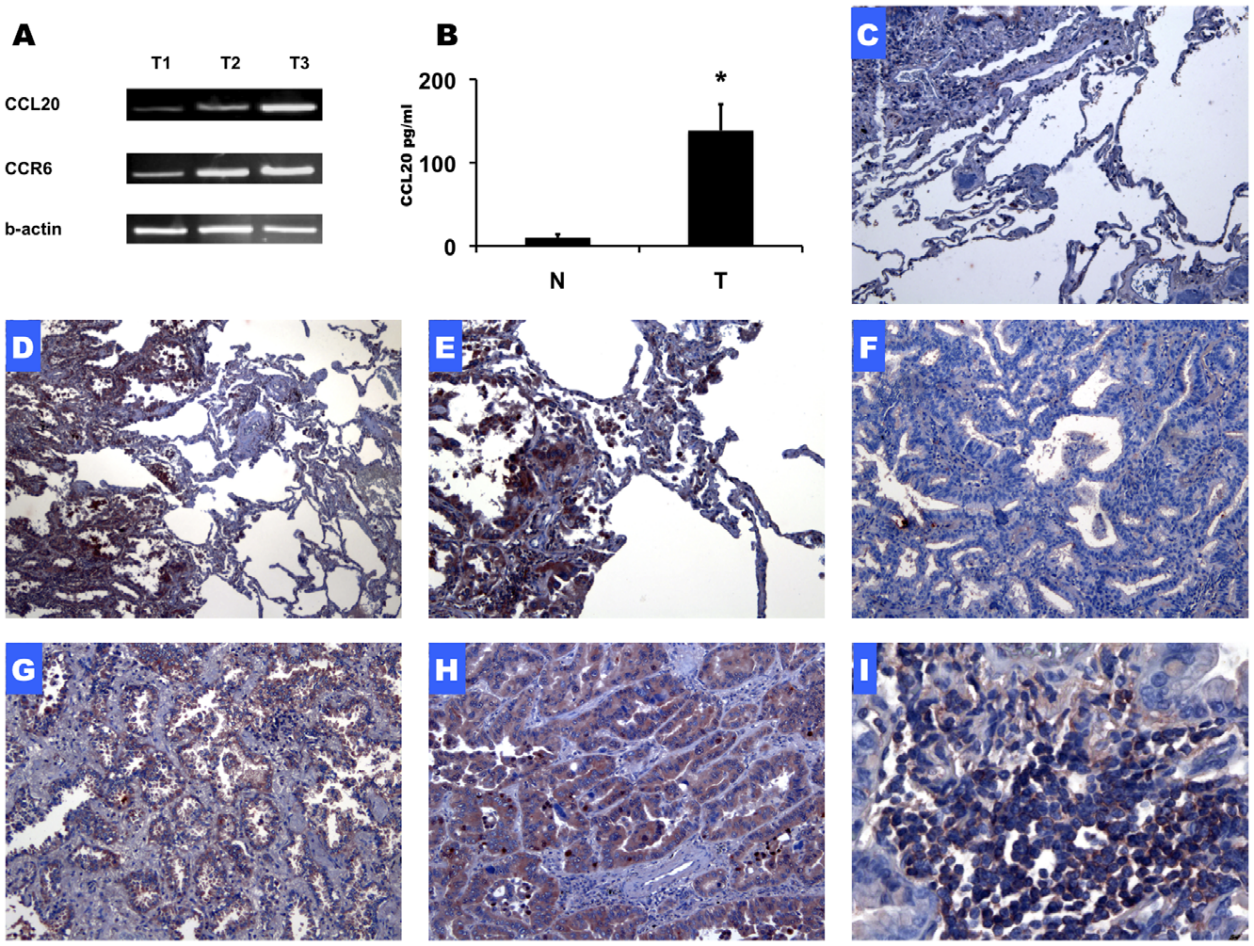
Expression of CCR6/CCL20 in NSCLC tissue samples. PCR signal for CCL20, CCR6 and beta-actin in three NSCLC tumor samples (A). CCL20 protein levels in NSCLC tumors and in tumor adjacent lung tissue (n = 3) (B). Representative immunohistochemistry staining for CCL20 and CCR6 in lung adenocarcinoma tissue sections. CCL20 - Low X10 (D) and high power X20 (E) magnification. CCR6 - Low power (X10) (F, G) and high power (X20) (H) magnification. Immune cell infiltrates located with in lung adenocarcinoma tumor stain positive for CCR6 (X40) (I). Negative control staining (X10) is shown (C). (*P<0.05)

The expression of CCL20 and CCR6 in fresh NSCLC samples was assessed by PCR and ELISA assays. Figure 1A depicts three representative tumor samples, documenting PCR signal for the ligand and its receptor. CCL20 protein levels in NSCLC tumors were significantly higher as compared to the levels of the protein in tumor adjacent normal lung tissue (Figure 1B).

Using immunohistochemistry, we defined the expression patterns of CCL20 and CCR6 in lung adenocarcinoma. Low X10 and high power X20 magnification of CCL20 staining is shown in Figure 1D and Figure 1E. Tumor tissue stained positive for CCL20 while tumor adjacent lung tissue was negative. Three samples of tumor sections stained for CCR6 are shown: in Figure 1F (X10), no CCR6 staining was observed. Figure 1G (X10) represents low to medium expression of CCR6 by tumor cells and Figure 1H (X20) demonstrates high CCR6 expression; in these samples, some of the tumor cell nuclei also stained positive for CCR6. Immune cell infiltrates located within lung adenocarcinoma tumor stained positive for CCR6 (X40) (Figure 1I). Negative control staining (X10) is shown in Figure 1C.

### CCR6/CCL20 expression and NSCLC disease progression

**Figure 2:**
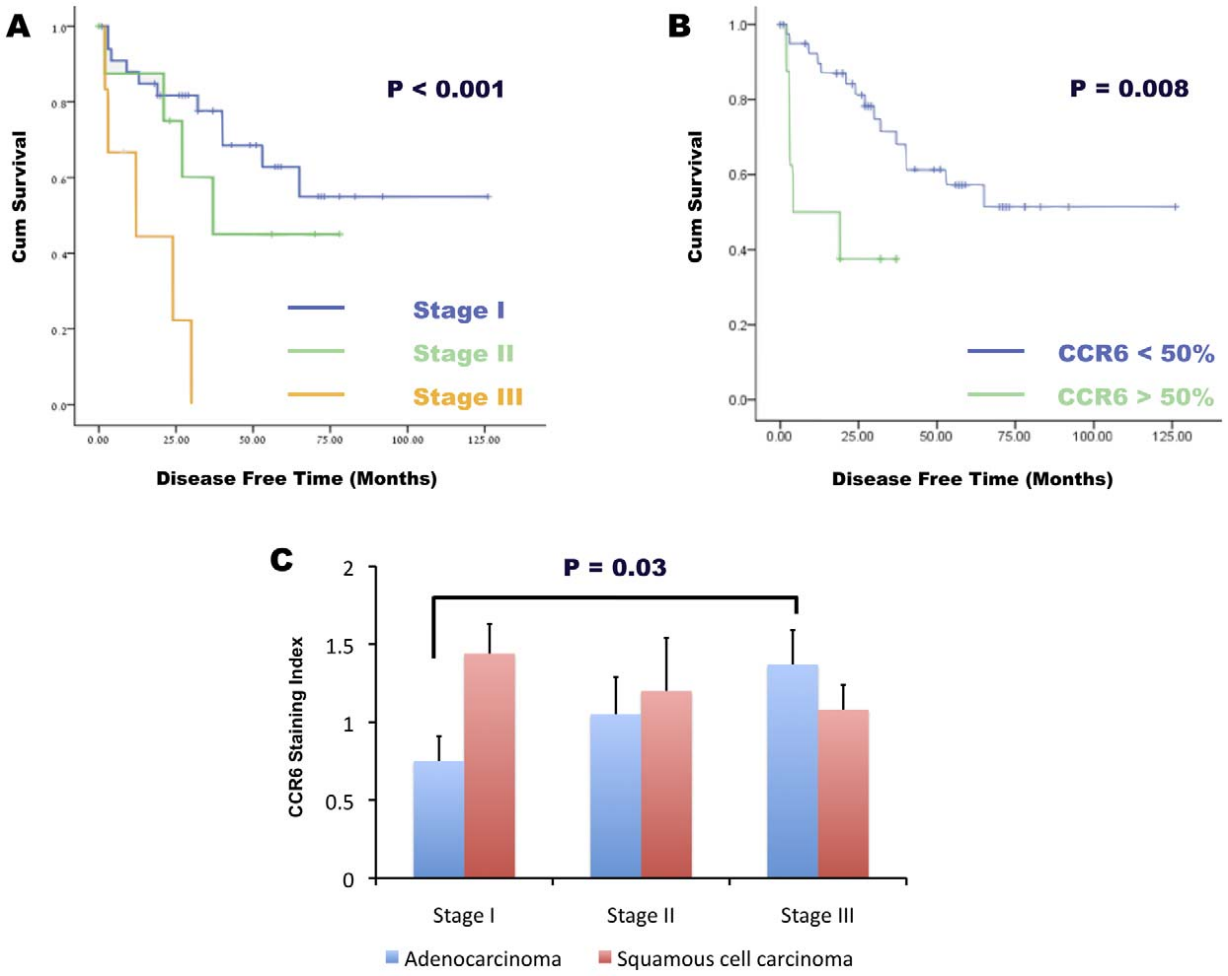
CCR6/CCL20 expression and NSCLC disease progression. Kaplan-Meier analysis of disease free survival interval (months) according to disease stage. (A). Kaplan-Meier analysis of disease free survival interval (months) according to percentage of CCR6 positive cells. (B). CCR6 staining index for lung adenocarcinoma and squamous cell carcinoma according to disease stage is shown (C).

To study the clinical relevance of CCL20/CCR6 expression in NSCLC, we tested the expression of the chemokine and receptor in 49 lung adenocarcinoma tissue samples removed during surgery. Results were correlated with patient pathological stage of disease and with time of disease recurrence. Patients' characteristics are shown in Table 1. Table 2 (for CCL20) and Table 3 (for CCR6) show the number of slides that were allocated to each staining category according to disease stage. The majority of tumor samples (38/49 (78%)) showed high (>50%) expression of CCL20. Oppositely, a minority of tumor samples (8/49 (16%)) expressed high (>50%) levels of CCR6. As expected, the Kaplan-Meier analysis indicated that a more advanced disease stage is associated with a shorter disease free survival (P<0.0001) (Figure 2A). Using the same method, we found that high CCR6 expression is associated with a shorter disease free survival (P = 0.008). COX regression analysis identified high CCR6 expression as a disease-stage independent marker for disease recurrence. High CCR6 expression conferred a 4.87-fold increased risk for disease recurrence (P = 0.0076, CI 95% 1.52–15.563) (Figure 2B).

**Table 1:** Patient's characteristics.

N =	49
Age	65.8 +/− 8.91
Gender	25 female/24 male
Stage I	32
Stage II	9
Stage III	8
Follow up	
Mean	54 +/− 23.5 months
Median	52 months
Range	18–125 months

**Table 2:** For CCL20, indicates the number of slides that were allocated to each staining category according to disease stage.

CCL20	0–25%	25–50%	50–75%	75–100%
Stage I	6	2	5	19
Stage II	1	0	2	6
Stage III	2	0	2	4
Total	9	2	9	29

Samples were divided into four categories according to percent of tumor cells staining positive: 1. 0–25% of tumor cells stained positive, 2. 25–50% of tumor cells stained positive, 3. 50–75% of tumor cells stained positive, 4. 75–100% of tumor cells stained positive.

**Table 3:** For CCR6, indicates the number of slides that were allocated to each staining category according to disease stage.

CCR6	0–25%	25–50%	50–75%	75–100%
Stage I	19	7	3	3
Stage II	9	0	0	0
Stage III	6	0	0	2
Total	34	7	3	5

Samples were divided into four categories according to percent of tumor cells staining positive: 1. 0–25% of tumor cells stained positive, 2. 25–50% of tumor cells stained positive, 3. 50–75% of tumor cells stained positive, 4. 75–100% of tumor cells stained positive.

### CCR6 expression and NSCLC disease stage

To examine the correlation between CCR6 expression and NSCLC disease stage, we measured CCR6 staining index in a tumor tissue array enclosing lung adenocarcinoma samples homogenously spread among the different disease stages. The array included 52 samples of lung adenocarcinoma (19 samples of stage I disease, 16 samples of stage II disease, 16 samples of stage III disease and 1 sample of stage IV disease) and 41 samples of squamous cell carcinoma (20 samples of stage I disease, 6 samples of stage II disease and 17 samples of stage III disease). The average CCR6 staining index was 0.75 +/− 0.16 for stage I adenocarcinoma, 1.05 +/− 0.24 for stage II adenocarcinoma and 1.37 +/− 0.22 for stage III adenocarcinoma. The increased CCR6 staining index observed in stage III relative to stage I adenocarcinoma was statistically significant P = 0.03 (Figure 2C). The average staining indices for squamous cell carcinoma were 1.44 +/− 0.19, 1.2 +/− 0.34 and 1.08 +/− 0.16 for disease stages I, II, III respectively; no significant statistical difference was found among this group (Figure 2C).

### Expression of CCR6/CCL20 in NSCLC-derived cell lines

**Figure 3:**
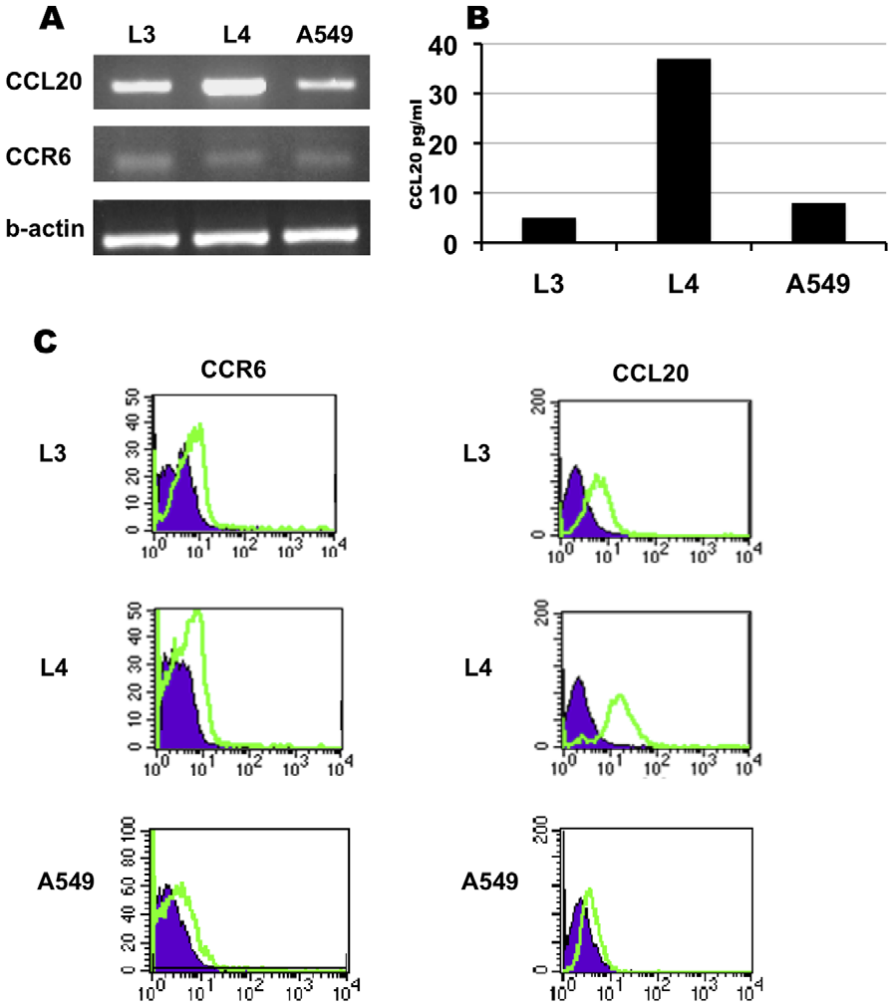
Expression of CCR6/CCL20 in NSCLC-derived cell lines. PCR signal for CCL20, CCR6 and beta-actin in L3, L4 and A549 cell lines (A). ELISA assay for CCL20 in the supernatant of L3, L4 and A549 cell lines (B). Flow Cytometry histogram analysis of CCR6 and CCL20 staining of L3, L4 and A549 cell lines, control antibody – purple, staining antibody – green line (C).

We characterized the expression of CCL20/CCR6 in three NSCLC-derived cell lines (L3, L4 and A549). A low level of CCR6 expression was detected in all three-cell lines (Figure 3A PCR and 3C flow cytometry). Differential expression and production of CCL20 by the three cell lines was detected (Figure 3A PCR, 3B ELISA and 3C Flow cytometry).

### CCL20 induces NSCLC proliferation and ERK phosphorylation

**Figure 4:**
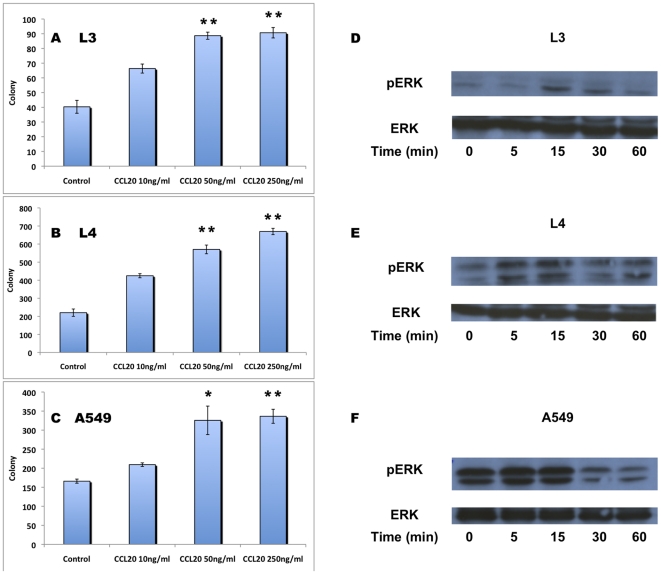
CCL20 induces NSCLC proliferation and ERK phosphorylation. Colony formation by L3, L4 and A549 cells in response to stimulation with increasing concentrations of CCL20 (A, B, C). ERK phosphorylation in L3, L4 and A549 cells in response to CCL20 stimulation. Phosphorylated ERK upper panel and total ERK lower panel (D, E, F). (*P<0.05, **P<0.01)

**Figure 5:**
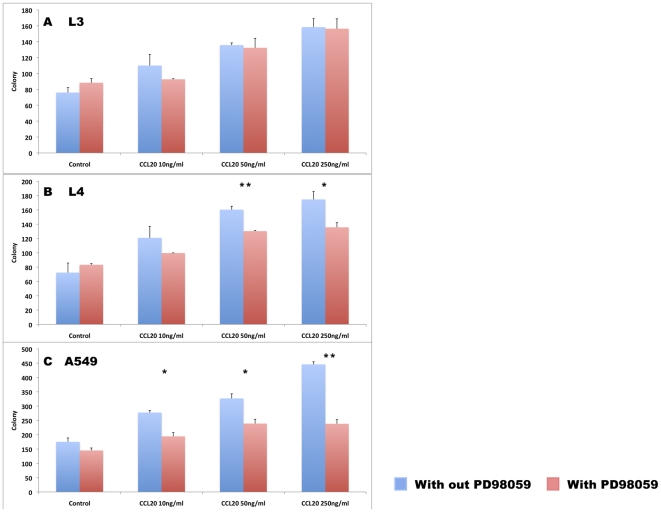
ERK inhibition decreases CCL20-mediated NSCLC proliferation. Colony formation by L3, L4 and A549 cells in response to stimulation with increasing concentrations of CCL20 in the presence or absence of the ERK inhibitor PD98059 (A, B, C).

The effect of CCL20 stimulation on neoplastic cell proliferation was studied using colony assays. Stimulation of L3, L4 and A549 cells by increasing concentrations of CCL20 promoted colony formation in a dose-dependent fashion (Figure 4A, B, C). CCL20 stimulation of L3, L4 and A549 cells induced ERK phosphorylation 5, 15 and 30 minutes after exposure (Figure 4D, E and F upper panel) with no change in total ERK levels (lower panel). Inhibition of phospho-ERK signaling by the specific inhibitor PD98059 did not affect the basal colony formation capacity of these cells (Figure 5A, B, C); however, it significantly decreased the potential of CCL20 to induce colony formation by L4 and A549 cells (Figure 5B, C).

### IL-17 is expressed in NSCLC and induces CCL20 production by NSCLC-derived cell lines

**Figure 6:**
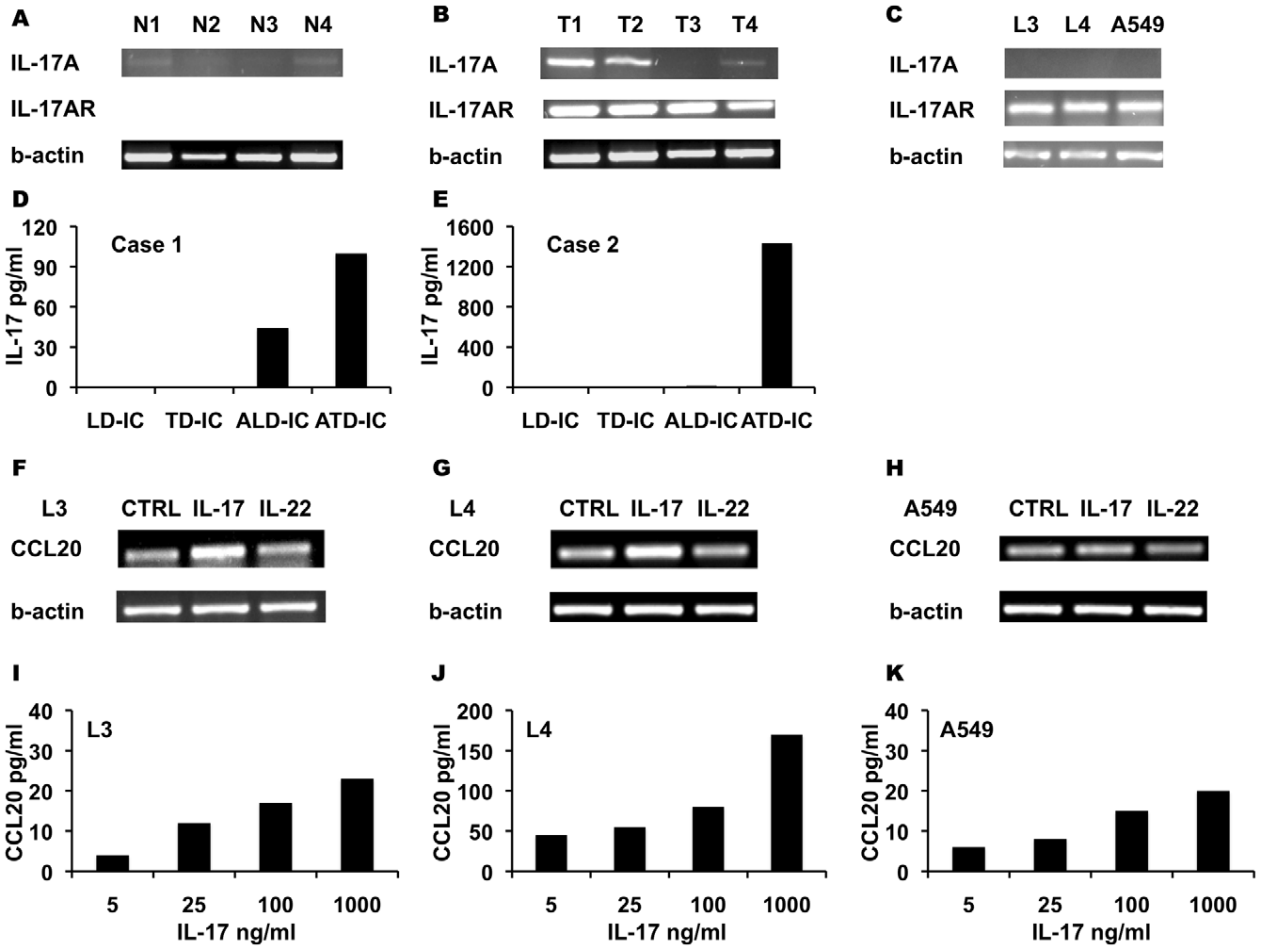
IL-17 is expressed in NSCLC and induces CCL20 production by NSCLC-derived cell lines. PCR signal for IL-17A, IL-17A receptor and b-actin in NSCLC tissue samples, in tumor adjacent lung tissue and in L3, L4 and A549 cell lines. Tumor adjacent lung tissue (A). NSCLC tumor sample (B). L3, L4 and A549 cell lines (C). Infiltrating immune cells were isolated from tumor tissue (TD-IC) and tumor adjacent lung tissue (LD-IC). Immune cells were incubated with or without anti CD3 antibody and production of IL-17 assessed by ELISA - two cases (D, E). Semi-quantitative PCR analysis for CCL20 in L3, L4 cells 549 cells in response to IL-17 and IL-22 stimulation (F, G, H). ELISA assays for CCL20 in the supernatant of L3, L4 cells 549 cells following stimulation with increasing concentrations of IL-17 (I, J, K).

The expression of IL-17A and IL-17A receptor in NSCLC tissue samples in tumor adjacent lung tissue and in L3, L4 and A549 cell lines was tested. PCR signal for IL-17A was low in two samples and negative in an additional two samples of tumor adjacent lung tissue (Figure 6A). Two out of four tumor samples showed high signal for IL-17A and one showed a weak signal (Figure 6B). The IL-17A signal was negative in all cancerous cell lines (Figure 6C). The IL-17A receptor was highly expressed by tumor samples and tumor cell lines (Figure 6B, C). In two cases, infiltrating immune cells were isolated from tumor tissue and tumor adjacent lung tissue; these cells were incubated with or without anti CD3 antibody (OKT3) and the production of IL-17 was assessed by ELISA. Activated tumor-derived immune cells produced higher levels of IL-17 (Figure 6D, E). The effects of IL-17 and IL-22 stimulation on CCL20 production by neoplastic cells were studied. Semi-quantitative PCR analysis showed that IL-17, but not IL-22, stimulation induced up-regulation of CCL20 mRNA in L3 and L4 cells and to a lesser extent in A549 cells (Figure 6F, G, H). ELISA assays showed a dose-dependent increase in CCL20 production following IL-17 stimulation (Figure 6I, J, K).

## Discussion

Chemokine receptor expression by tumor cells enhances their survival, proliferation and metastatic potential. Both tumor cells and tumor stroma produce chemokines [8]. Several groups examined the role of the chemokine/chemokine receptor pair, CCL20/CCR6, in malignancy. Pirus Ghadjar et al., have shown that CCR6 expression guide hepatic metastasis of colonic malignancies [24]. Kimsey et al., reported that co-localization of CCL20 and CCR6 promotes pancreatic cancer cell invasion [25]. Others and we have shown that CCL20/CCR6 auto-signaling occurs in prostatic cancerous cells and that tumor CCR6 expression correlates with disease aggressiveness [7], [26], thus marking intra-tumoral CCL20/CCR6 interactions as a pro-carcinogenic axis. CCL20 is over-expressed by lung epithelial cells in response to smoke and particulate matter [27]. The role of this chemokine in lung cancer pathogenesis has not been studied thus far. To test the potential pro-carcinogenic effects of CCL20 and CCR6 in lung cancer, we first aimed to characterize the expression and tissue localization of this chemokine/chemokine receptor pair in NSCLC tumors. Immunohistochemistry studies indicated that the majority of lung adenocarcinoma tumor samples highly stained for CCL20, while only a minority highly expressed CCR6. Using Kaplan-Meier analysis, we looked for associations between the clinical course of lung adenocarcinoma patients and the extent of CCL20/CCR6 staining in their tumor samples. We found that high CCR6 expression was associated with a shorter disease-free survival (P = 0.008). Furthermore, Cox regression analyses showed that high CCR6 expression was associated with a 4.87-fold increased risk for disease recurrence (P = 0.0076, CI 95% 1.52–15.563) and the effect of CCR6 was independent of pathological stage of disease. The strength of this study lies on focusing on an accurate pathological staging, a specific diagnosis (lung adenocarcinoma) and the long duration of clinical follow-up. The weakness of this study is its relatively small sample size (49 patients). In order to expand the sampled data, we performed an additional analysis of CCR6 expression using a tumor tissue array enclosing over 50 lung-adenocarcinoma samples homogenously spread among the different disease stages. In this array, we found a higher CCR6 staining index among advanced stage adenocarcinomas. This pattern was unique for adenocarcinoma, albeit not detected in squamous cell tumors. Shedden K. et al., have extensively examined the gene expression-based survival of lung adenocarcinoma patients [28]. Their data, containing clinical parameters and gene profiling of over 400 samples from lung adenocarcinoma patients, is available for download from the NCBI. We examined the expression patterns of CCL20 and CCR6 in this database. The reported gene array signal detection was low for CCR6, ranging between 4 to 115 and high for CCL20, ranging from 2–5,597. In contrast to the immunohistochemistry results described above we have not found a correlation between CCR6 gene array signal intensities and lung adenocarcinoma disease parameters. The different methods of detection, degradation of CCR6 mRNA and reduced sensitivity of the specific probe for CCR6 may account for this discrepancy. Interestingly however, we have found highly significant correlations between CCL20 signal intensities and three clinical parameters: 1) Vital status of patients (Alive – 535+/−58 vs. dead – 786 +/− 72, P = 0.008), 2) Tumor differentiation (Well differentiated – 334+/−83 vs. poorly differentiated – 793 +/− 88, P = 0.004) and 3) T score of TNM staging system (T-1 – 552+/−68 vs. T-3/4 – 1055 +/− 207, P = 0.005). Take together, this data suggest that CCL20/CCR6 interactions in the tumor microenvironment may stimulate NSCLC disease progression. CCR6 is identified as a potential disease marker. These observations add to the accumulating clinical data regarding the involvement of CCL20/CCR6 in malignancy and mark this axis as a potential therapeutic target.

To test in vitro the potential effects of CCL20/CCR6 interactions in NSCLC, three tumor cell lines were tested. L3, L4 and A549 cells expressed low levels of CCR6 and produced varying levels of CCL20. Stimulation of these lines with CCL20 induced colony formation in a dose-dependent manner. CCR6-dependent ERK phosphorylation mediated proliferation of colorectal cancer cells [29]. Activation of ERK signaling was also considered as a key pathway in NSCLC development [30]. We found that CCL20 induced ERK phosphorylation in L3, L4 and A549 cells. Furthermore, inhibition of ERK signaling by PD98059 decreased the potential of CCL20 to induce colony formation while it did not affect the basal numbers of colonies formed, thus demonstrating the pro-proliferative potential of CCL20/CCR6 auto-signaling in NSCLC and marking ERK as an intracellular mediator of these signals.

Autocrine and paracrine chemokines/chemokine receptor interactions link chronic inflammation to malignancy [31]. CCL20 is a bi-functional peptide with both innate and adaptive immune properties. CCL20 is induced in human airway epithelia by various pro-inflammatory signals as well as by smoke and particulate matter [15], [32]. CCL20 is the only chemokine known to interact with the receptor CCR6 [32]. CCL20 promotes smoke- related lung inflammation by the recruitment of CCR6-expressing subset of immune cells into lung parenchyma [16], [17]. For example, chronic cigarette smoke exposure induced the accumulation of CD4+ CCR6+ Th17-type in mouse airways resulting in airspace enlargement [33], [34]. Numasaki et al. reported that NSCLC tumors contain infiltrates of CD3+ IL-17 expressing cells and suggested that the crosstalk among these cells and the neoplastic cells enhances local tumor progression [35]. The trafficking of Th-17 cells is guided by CCR6 [36]. Here we show that intra-tumoral levels of CCL20 were elevated as compared to tumor adjacent lung tissue and that CCR6 positive immune cell infiltrates were found in the tumor parenchyma. PCR signal for IL-17 was detected in three out of four NSCLC tissue samples, however it was not found in any of the tumor cell lines and was only weakly expressed in two out of four tumor adjacent lung tissue samples. Activation of tumor infiltrating immune cells with anti CD3 antibody induced IL-17 production. In line with previous reports, these observations suggested that IL-17 producing cells infiltrate NSCLC tumors. All NSCLC tumors and cell lines expressed the IL-17 receptor. We found that stimulation of NSCLC cell lines with IL-17- induced CCL20 production in a dose-dependent manner. Immature dendritic cells, FoxP3 regulatory T cells and Th-17 cells all expressed CCR6 and were reported to accumulate in NSCLC tumors [20], [36], [37]. We speculate that IL-17-induced CCL20 production in the tumor microenvironment may act in an autocrine manner to stimulate tumor cell growth and in a paracrine manner to enhance the tumor-associated inflammatory response.

In conclusion, our findings suggest that the CCL20/CCR6 axis promotes NSCLC disease progression. CCR6 is identified as a potential new prognostic marker and the CCL20/CCR6/IL-17 axis as a potential new therapeutic target. Larger scale studies are required to consolidate these observations.

## Supporting Information

Figure S1 **Fingerprinting according to reference 22.**
(TIFF)

Figure S2 **To demonstrate the specificity of anti-CCR6 clone 53103.11 we incubated fresh PBMC in the presence or absence of 20ug/ml CCL20 for one hour and stained the cells for CCR6 or with isotype control antibody.** Reduced CCR6 staining was observed in CCL20 treated cells. Grey - control antibody, Red - no CCL20 treatment, Black - CCL20 treatment.
(TIFF)
