# Supplementary figures and images for: Involvement of CCR6/CCL20/IL-17 Axis in NSCLC Disease Progression
Source: PLoS One. 2011 Sep 15;6(9):e24856. doi: 10.1371/journal.pone.0024856 (PMC3174223; doi:10.1371/journal.pone.0024856)

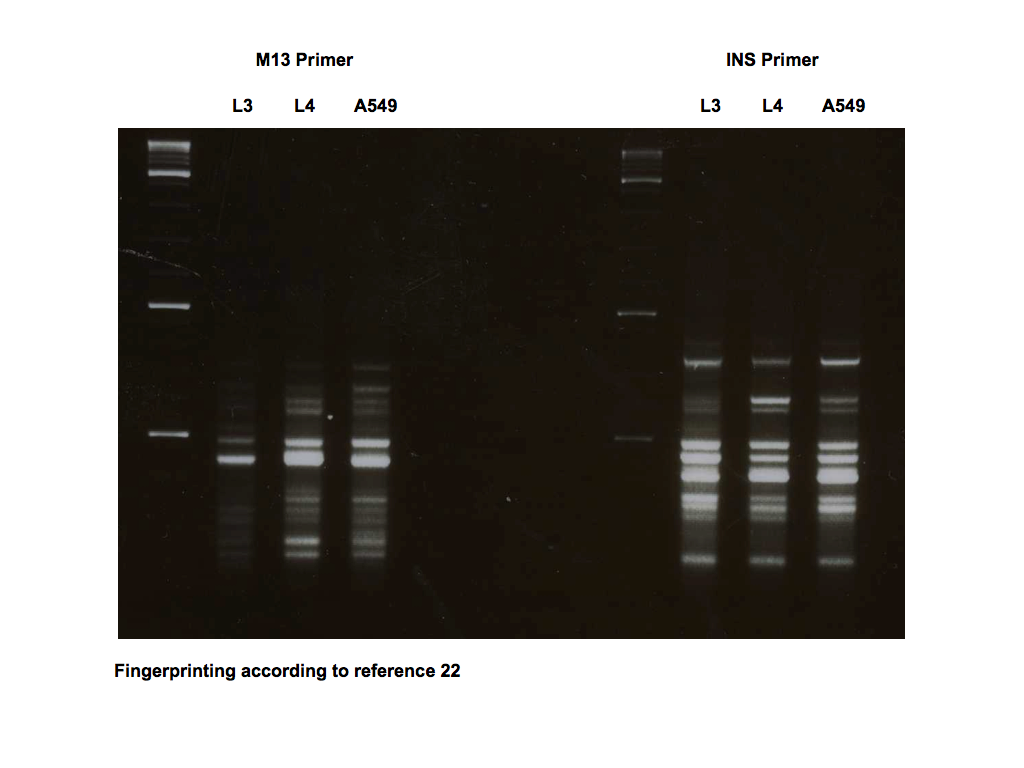

Supplement: Figure S1 — Fingerprinting according to reference 22. (TIFF) [file pone.0024856.s001.tiff]

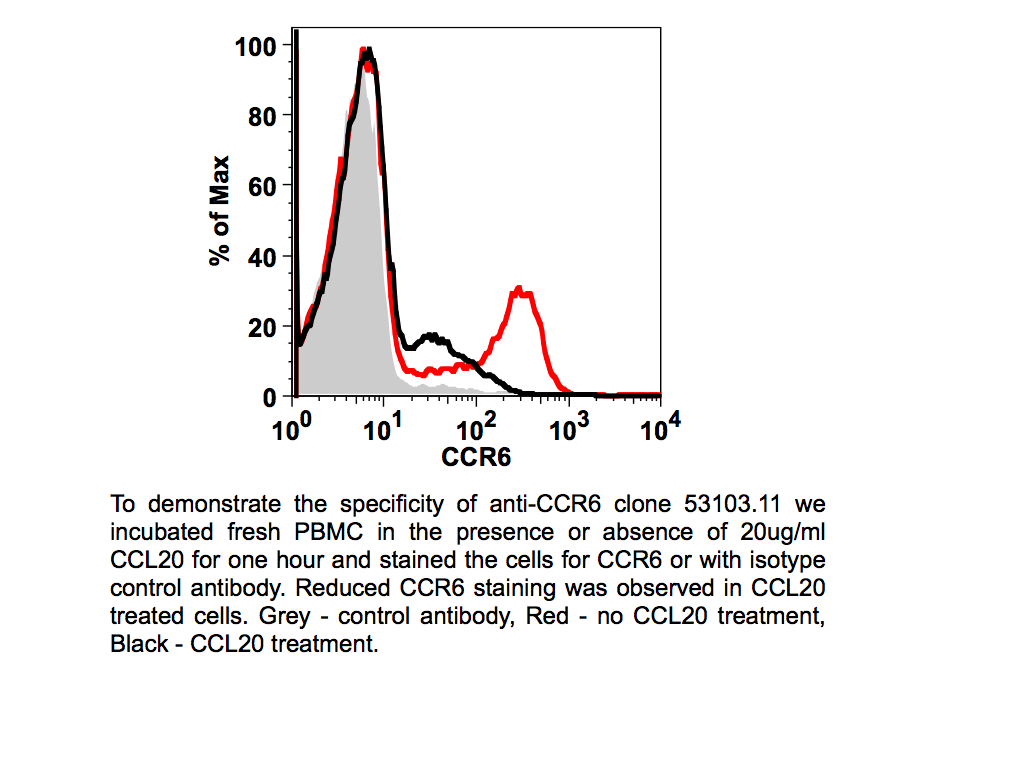

Supplement: Figure S2 — To demonstrate the specificity of anti-CCR6 clone 53103.11 we incubated fresh PBMC in the presence or absence of 20ug/ml CCL20 for one hour and stained the cells for CCR6 or with isotype control antibody. Reduced CCR6 staining was observed in CCL20 treated cells. Grey - control antibody, Red - no CCL20 treatment, Black - CCL20 treatment. (TIFF) [file pone.0024856.s002.tiff]
